# Sleep Disturbance, Sleep Disorders and Co-Morbidities in the Care of the Older Person

**DOI:** 10.3390/medsci9020031

**Published:** 2021-05-21

**Authors:** Christine E. Mc Carthy

**Affiliations:** 1 Department of Geriatric Medicine, University Hospital Galway, Galway, Ireland; C.MCCARTHY54@nuigalway.ie; 2 HRB-Clinical Research Facility, National University of Ireland, Galway, Co., Galway, Ireland

**Keywords:** sleep, sleep disorders, older patient, geriatric medicine, comprehensive geriatric, assessment, co-morbidity, co-morbidities

## Abstract

Sleep complaints can be both common and complex in the older patient. Their consideration is an important aspect of holistic care, and may have an impact on quality of life, mortality, falls and disease risk. Sleep assessment should form part of the comprehensive geriatric assessment. If sleep disturbance is brought to light, consideration of sleep disorders, co-morbidity and medication management should form part of a multifaceted approach. Appreciation of the bi-directional relationship and complex interplay between co-morbidity and sleep in older patients is an important element of patient care. This article provides a brief overview of sleep disturbance and sleep disorders in older patients, in addition to their association with specific co-morbidities including depression, heart failure, respiratory disorders, gastro-oesophageal reflux disease, nocturia, pain, Parkinson’s disease, dementia, polypharmacy and falls. A potential systematic multidomain approach to assessment and management is outlined, with an emphasis on non-pharmacological treatment where possible.

## 1. Introduction

Sleep, which has an important role in human health, is commonly disrupted in those aged ≥65 years [1,2,3,4]. This disruption has been previously described as a multifaceted geriatric syndrome [5]. Given that sleep impairment is associated with increased risks of mortality, cardiovascular disease, impaired quality of life (QOL) and falls, it should be a target of holistic patient management, and not considered a normal phenomenon of ageing [6,7,8,9]. In this article, the author discusses the potential causes of sleep impairment in older age, in addition to an approach to its assessment and management, with specific sections on sleep disorders and co-morbidities.

## 2. What Disturbs Sleep in the Older Patient

While not a consequence of healthy ageing, older patients are at a higher risk of sleep impairment than their younger counterparts [10,11]. Typical changes in sleep architecture and continuity seen in older age include reduction in slow wave sleep (SWS), percentage of rapid eye movement (REM) sleep, total sleep time (TST), sleep efficiency (proportion of time in bed spent asleep) and an increase in sleep onset latency (time to fall asleep) [12]. Older people can also experience phase advance in their internal sleep-wake cycle (circadian rhythm), going to bed and getting up earlier [13]. The reason for this susceptibility is not yet fully determined. It is likely a multifactorial phenomenon, with intrinsic and extrinsic factors exerting influence. (See Table 1).

From a neurological and endocrinal perspective, there are a lot of potential factors that can impair sleep. Neurotoxin deposition, age related blood brain barrier dysfunction and local small vessel disease may cause grey and white matter loss, disrupting sleep spindle and SWS generation. In addition, neurotransmitter dysregulation may directly impair sleep drive, and neurodegeneration in the suprachiasmatic nucleus can impact the normal circadian rhythm [14]. Accumulation of lens pigmentation and loss of certain retinal ganglion cells may reduce light transmission and also negatively influence circadian rhythm [15,16]. Secretion of certain hormones, such as cortisol and growth hormones that have a circadian rhythm or are secreted during certain sleep phases, may also change with ageing, with a potential direct or indirect effect on sleep [13].

Life events, such as bereavement and loss, are more common in older age, and are also associated with sleep problems [17,18]. In addition, low levels of physical activity and social engagement in older adults have been associated with sleep impairment [19,20,21].

The prevalence of primary sleep disorders, such as insomnia, sleep disordered breathing (SDB) and REM sleep are also common in older people [4,22,23,24]. In addition, increasing chronic conditions in older life and medication numbers are associated with impaired sleep [21,25,26]. The relationship in some cases may be bi-directional, with insufficient sleep being both a consequence of and risk factor for disease development [27].

## 3. Sleep Disorders and the Older Patient

### 3.1. Insomnia

Insomnia is common in older age, although reported prevalence varies [28]. Diagnosis requires persistent difficulty with sleep and secondary daytime dysfunction, despite adequate sleep opportunity [29]. The diagnosis is made clinically, through careful history taking and exclusion of other sleep disorders. Where another sleep disorder is suspected, such as sleep disordered breathing (SDB), referral for polysomnography (PSG) should be considered [27,30]. Subjective measures, such as sleep diaries and rating and screening scales, may also assist the diagnosis, but it should be noted that many have not been validated in the older population or in patients with cognitive impairment [31,32,33].

Where possible, non-pharmacologic therapy should be considered as first-line treatment. A recent systematic review of sleep hygiene education (SHE) for treatment of insomnia found that SHE was associated with sleep improvements [34]. Cognitive Behavioural Therapy for Insomnia (CBT-I) was the more effective option in the same systematic review, and appears to significantly improve symptoms in older patients [35,36]. In one randomised controlled trial (RCT) of older patients with insomnia, in fact, CBT-I appeared superior to zopiclone for both short and long-term management [37]. One significant barrier to CBT-I, however, is access to therapists [38]. Given this, digital CBT-I (dCBT-I) is an emerging possibility in the older patient cohort [39]. Relaxation and mindfulness techniques also have a role in treatment [34,36]. Results vary or are limited for other non-pharmacological approaches [40,41].

Pharmacological management of insomnia in older patients should be undertaken with absolute caution. While statistically significant effects on sleep continuity have been seen with hypnotic use, the magnitude of the effects are small, and associated risks are both significant and clinically relevant [42]. These risks include falls, hip fractures, impairment of cognitive function and higher overall mortality [43,44,45,46,47]. CBT-I should generally be considered as first-line treatment for chronic insomnia in older patients [48]. Abad and Guilleminault recommended that hypnotic medications may be considered as adjunctive therapy initially if insomnia symptoms are severe, impacting activities of daily living (ADL) or QOL [49]. A short course with a plan for discontinuation is recommended, with short-acting drugs preferred and choice of agent dependent on the predominant insomnia symptom. Benzodiazepines are not recommended initially, and should only be considered as a second-line option [49]. Consideration of co-morbidities such as depression and their management, in addition to medication review, is also an important element of care. Melatonin may also be helpful, particularly when sleep onset insomnia symptoms predominate, in older patients [50,51,52].

### 3.2. Sleep Disordered Breathing

SDB is the umbrella term used to describe many disorders of the respiratory pattern occurring periodically during sleep, including obstructive sleep apnoea (OSA) and central sleep apnoea (CSA) [53]. The incidence of SDB increases with age, with a wide variety of prevalence reported in older adults [54]. Patients with OSA frequently stop breathing during sleep due to obstruction of the upper airway, while CSA is characterised by apnoeic events in the absence of respiratory effort [55]. OSA has been associated with obesity, diabetes, hypertension (HTN) and many other co-morbidities, while CSA is more commonly associated with left ventricular failure, opioid use and neurodegenerative disease [53,56,57]. It has been suggested that the phenotype of OSA differs between older and younger adults, with worsening of the upper airway anatomy having a more significant role to play in older patients, in addition to a weaker association with obesity [58,59].

The typical presentation of OSA in a younger patient of nocturnal snoring, choking, nocturia and excessive daytime sleepiness does not necessarily occur in the older patient [60,61]. Screening tools may be helpful in the geriatric clinic, however, again, there is limited literature in relation to validation of these tools, with the vast majority of studies being conducted with participants of a mean age of <65 years [62]. PSG is the gold standard diagnostic tool, however, the use of portable or home-based devices is increasing [53,63]. While the majority of research in these devices was also conducted in patients with a mean age of <65 years, they have been studied in geriatric populations, and they do appear to be effective for diagnosis in patients at high risk of the disorder [63,64,65].

SDB has been associated with stroke, nocturnal hypertension, glaucoma, falls and mortality in the older population [66,67,68,69,70,71,72]. A significant association has also been observed between SDB and cognitive impairment [73]. Given this, treatment of SDB should be considered important in older patients [74]. Positive airway pressure therapy (PAP) is the gold standard of care, and there is some evidence to support its use in older populations, where it has been associated with a reduction in falls, improved cognition, improved cardiovascular outcomes, improved outcomes post-stroke and improved QOL [70,75,76,77,78,79,80,81,82]. In addition, age alone does not seem to impact PAP adherence, and reasonable adherence has also been observed in patients with mild to moderate Alzheimer’s dementia (AD) [83,84]. Minimisation of sedative medications in addition to optimising the management of other comorbidities are also key areas to consider [53,54,74].

### 3.3. Restless Leg Syndrome

Restless leg syndrome (RLS) is a movement disorder characterised by an urge to move the limbs in response to an uncomfortable sensation [85]. The lower limbs are most commonly affected, with a nocturnal predominance of symptoms [86]. Screening questions such as: “When you try to relax in the evening or sleep at night, do you ever have unpleasant, restless feelings in your legs that can be relieved by walking or movement?” may be helpful, and have been studied in patients with a mean age of >60 years [87]. There are several mimics of RLS, including leg cramps, positional discomfort, leg oedema, neuropathy, myalgia and arthritis, and use of a diagnostic criteria should establish the diagnosis, such as the International Restless Legs Syndrome Study Group (IRLSSG) diagnostic criteria [88]. Reported community prevalence ranges between 1.9% and 15%, generally increasing with age [89]. Dysfunction of the dopaminergic system and iron deficiency are thought to be involved in its pathogenesis [90,91]. RLS is also associated with many other common co-morbidities, including cardiovascular disease, hypertension, diabetes and depression [92,93,94].

Sleep symptoms are common in RLS, with patients reporting an inability to fall or stay asleep [95]. Associations with daytime sleepiness, fatigue and issues with daytime functioning have also been reported [96]. In two large cross-sectional studies, sleep symptoms were reported as the “most troublesome” symptom by more than one-third of participants [95,97]. Severity of symptoms in older patients has also been linked to poorer QOL, social and daily functioning and emotional well-being [98].

Evidence based non-pharmacological therapy for RLS is scarce. A recent systematic review found that exercise, counter-strain manipulation, compression devices, repetitive transcranial magnetic stimulation, infrared therapy, acupuncture, vibration pads and yoga were associated with improved outcomes [99]. Studies were deemed not of high quality in this systematic review, however, with few identified, and the majority of participants were <65 years old [99]. Consideration of potentially exacerbating medications may be helpful, with some serotonergic antidepressant medications implicated, and bupropion potentially helpful [100]. The evidence, however, is very limited, and medication review should be individualised [100,101].

Obtaining serum ferritin levels in patients with RLS should be considered, as replacement of iron in deficient patients can improve symptoms [102,103,104]. The American Academy of Neurology recommends considering ferrous fumarate supplementation in combination with vitamin C in patients with RLS and serum ferritin of <75 mcg/L, and considering IV iron replacement if severe symptoms are present [85]. In the setting of iron deficiency anaemia in the older patients, other specific considerations should be undertaken, including the impact of chronic disease on ferritin level, work up for cause and the tolerance of oral supplementation [105].

Pharmacological options, including dopamine agonists, alpha-2 delta calcium channel ligands and levodopa, should be considered in cases where sleep, QOL or daytime functioning is impaired [85]. Opioids and benzodiazepines may also be helpful, but caution should be undertaken due to their side effect profile, to which older patients are particularly susceptible [85,106,107]. In relation to subjective sleep complaints, there is some evidence to support the use of ropinirole, pramipexole, rotigotine, gabapentin enacabril, pregabalin and levodopa [85,108,109,110,111,112,113,114,115,116]. It should be noted, however, that the mean age was <60 years in these studies [108,109,110,111,112,113,114,115,116]. The author recommends considering the individual patient in the context of potential side effects and medication interactions prior to considering therapeutic options.

### 3.4. REM Sleep Behavioural Disorder

REM sleep behaviour disorder (RBD) is a parasomnia characterised by the lack of paralysis during REM sleep, resulting in patients “acting out their dreams”. Unpleasant and vivid dreams are also a common symptom [117,118]. It is a disorder of older age, typically diagnosed between the ages of 50–85 years [118]. A recent population-based study of 1977 adults between the ages of 40–80 years estimated the prevalence of RBD at 1.06% [119].

RBD has been associated with poorer QOL in addition to poorer subjective sleep quality in comparison to healthy controls [120]. Sleep-related injuries to the patient and/or their bed partner may also occur [121,122]. Many patients with RBD are also subsequently diagnosed with a neurodegenerative disorders of synuclein aggregation, such as Parkinson’s disease (PD), dementia with Lewy bodies (DLB) and multiple system atrophy (MSA) [123,124]. A recent prospective cohort study of 1280 patients with RBD found an overall annual conversion rate to neurodegenerative disease of 6.3%, with 73.5% conversion over 12 years of follow-up [125]. RBD likely represents a prodromal phase of the disease in these instances, but it is yet to be determined whether the sleep disturbance is entirely secondary to the ongoing neurodegenerative process, or contributes to the disease pathogenesis [126].

RBD’s definitive diagnosis requires PSG [29]. The single screening question in older patients—“Have you ever been told, or suspected yourself, that you seem to ‘act out your dreams’ while asleep (for example, punching, flailing your arms in the air, making running movements, etc.)?”—may be useful, in addition to interviewing the bed partner [118,127,128]. Differential diagnosis includes OSA, sleep walking, sleep terrors, nocturnal frontal lobe epilepsy, periodic limb movement, arousals associated with confusion, dissociative states and malingering [118].

No established guidelines exist in relation to the management of RBD, and large RCTs have not been undertaken [118,129]. The main goal of treatment currently is to prevent sleep-related injuries and dreams patients find disturbing. The decision to start pharmacotherapy should be made on an individual basis, and should not generally be considered unless there is injurious behaviour or significant sleep disruption [118,130]. Pharmacologic options include clonazepam and melatonin, but these recommendations are based predominantly on case series, prospective studies and expert opinions [118,131]. Recent small RCTs have not determined either medication efficacious, and larger RCT trials are needed [132,133,134].

Modification of the sleep environment, including removing dangerous items, placing the mattress on the floor and sleeping in separate beds, may help prevent injury in cases with injurious behaviour [135]. Consideration of other potential sleep disorders should also be undertaken. In addition, tricyclic and serotonergic antidepressants and lipophilic beta blockers may exacerbate RBD and should be considered for review in patients, if thought medically appropriate [129].

## 4. Common Co-Morbidities and Their Relationship with Sleep

### 4.1. Depression

Depression in the older population may present differently than in the younger population, and older patients with depression may be more likely to report sleep disturbance [136,137]. A bi-directional relationship likely exists in relation to sleep disturbance and depression, with sleep disturbance representing both a risk for, and symptom of, depression [138,139].

In younger patients with concomitant depression and insomnia, augmenting treatment for depression with CBT-I has been associated with improved depressive and sleep symptoms [140]. CBT-I has also been associated with improved depression and insomnia severity in older adults with both conditions, and should be considered in this group [141]. Medication review and pharmacological therapy should also be considered, when indicated. In patients with depression and insomnia, certain medications for depression may negatively impact sleep. Selective serotonin reuptake inhibitors (SSRIs) and serotonin-norepinephrine reuptake inhibitors (SNRIs), for example, have been associated with increased wakefulness, insomnia and reduced REM sleep time [142]. Sedative antidepressants may be preferable when conditions co-exist, however, the majority of research in this area has been undertaken in younger patients. In middle-aged patients, mirtazapine has been associated with reduced hypnotic co-prescription when compared to SSRIs [143]. Increased total sleep time and reduced sleep onset latency was observed in an open-label study of mirtazapine in six patients aged <65 years with depression [144]. Similar effects were seen with trazadone in 10 middle-aged patients with depression [145]. Agomelatine was also associated with improved sleep parameters in a similar study of middle-aged patients, and may be beneficial for older patients with depression and co-morbid insomnia [146,147].

### 4.2. Heart Failure

Patients with heart failure (HF) commonly complain of non-restorative sleep, increased sleep onset latency, night time awakenings and early morning wakening [148,149]. Insomnia and SDB are also more prevalent in HF patients than in the general population [150,151,152]. The relationship between HF and sleep is, again, likely bi-directional. Sleep disturbance is associated with several cardiovascular disease risk factors, and cardiovascular disease, in addition to higher sympathetic activity [153,154,155]. With this considered, sleep disturbance may lie along the causal pathway for forms of HF, or potentially exacerbate the condition. In contrast, nocturnal symptoms of HF, such as orthopnoea and paroxysmal nocturnal dyspnoea, may cause or exacerbate sleep disturbance [156]. The Rotterdam Study found that clinical heart failure, but not cardiac dysfunction alone, predicted a reduction in sleep quality in longitudinal analysis, implicating HF symptoms in sleep disturbance [157]. In addition, nocturia and diuretics in HF patients have been associated with impaired sleep [158,159]. Concomitant depression and mood disorders may also have a significant impact [156].

Optimising HF management may positively impact sleep quality, while diagnosis and treatment of underlying sleep disorders should also be considered. There is some evidence, for example, suggesting that angiotensin converting enzyme (ACE) inhibitors may positively impact sleep in heart failure patients [160,161]. Beta blockers may also positively impact CSA in HF [162]. In addition, manipulating the timing of diuretic therapy to avoid periods of sleep, with consideration of diuretic half-life, has been recommended [158,159,163].

### 4.3. Chronic Respiratory Disorders

Reduced sleep quality has been associated with chronic respiratory disorders, including chronic obstructive pulmonary disease (COPD) and asthma [164,165]. In this setting, a relationship between poor sleep quality and lower oxygen saturations has been observed [164,166]. Level of asthma control has also been associated with sleep quality and sleep duration [167,168,169]. Again, with respiratory disorders and sleep, relationships are likely bi-directional, with nocturnal symptoms of respiratory illnesses likely contributing to arousals, and sleep dysfunction potentially influencing lung function and inflammation [170,171].

Optimal treatment of patients with sleep problems and respiratory disorders is complex. Sleep and stages of sleep can affect breathing through multiple mechanisms, including reduction in skeletal muscle tone and reduced ventilatory response to chemical and mechanical stimulus, potentially leading to inadequate ventilation during sleep in compromised lungs [172]. The possibility of co-morbid SDB, hypoxia and hypercapnia should be considered [173]. One review suggests that referral for overnight pulse oximetry should be considered in patients with low daytime oxygen saturations (<93%), and referral for PSG if symptoms are suggestive of SDB in COPD [174]. It should be noted, however, that long-term oxygen therapy in COPD patients with moderate desaturation has not been shown to have an impact on disease outcomes including sleep quality [175].

Management should include optimisation of the underlying respiratory condition. Improved asthma control, for example, has been associated with improved sleep quality [169]. Medical optimisation is, however, complex. Some medications, such as theophylline and corticosteroids, may negatively impact sleep [176,177]. These findings, however, have not been consistent, and these medications may improve sleep in patients with respiratory disease [178]. An individual approach in the older population, with cognisance of medication side effects, should be undertaken, while considering the disease guidelines. Hypnotics should be avoided where possible, especially benzodiazepine medications, due to the risk of respiratory depression, while melatonin may be a promising potential treatment option [179,180,181,182].

### 4.4. Pain and Disorders Associated with Pain

Chronic pain and insomnia are closely associated, with 50.4% of chronic insomnia participants in one cross-sectional study reporting chronic pain [25]. The relationship is, again, likely bi-directional, with experimental evidence of pain being associated with night time arousals, and sleep deprivation being associated with increased spontaneous pain incidence [183,184]. Studies have also demonstrated that, in disorders associated with chronic pain, poor sleep is associated with subsequent increased pain, in addition to increased pain being associated with poorer subsequent sleep [185,186]. Active pain treatment has been associated with improved sleep quality, with varying effects dependent on medications used and setting [187,188]. CBT-I is also a promising area in relation to improving chronic pain in older adults with insomnia [189].

### 4.5. Gastro-Oesophageal Reflux Disease

Gastro-oesophageal Reflux Disease (GORD) has been associated with insomnia and impaired sleep quality and quantity, after adjusting for multiple confounders [190]. In one cross-sectional survey, 75% of patients with nocturnal GORD symptoms stated that symptoms affected their sleep, with 42% accepting that they could not sleep through the night, and 34% sleeping in a seated position [191]. Again, there is a possibility of a bi-directional relationship, with nocturnal GORD symptoms causing sleep arousals, and in turn, sleep disturbances exacerbating GORD via hyperalgesia and prolonged acid contact time [192]. Lifestyle interventions such as raising the head of the bed and avoiding late meal times may be beneficial [193]. Treatment with proton pump inhibitors may also be beneficial in selected patients, for both nocturnal GORD symptoms and associated sleep indices [194].

### 4.6. Nocturia

Nocturia may significantly effect sleep [195]. Severity of nocturia has been associated with poor sleep quality and reduced sleep duration [196,197]. Using objective measures, more frequent nocturia has also been associated with reduced SWS, which is thought to have an important role in cerebral restoration and recovery [198,199]. In one cross-sectional study of older patients, nocturia was found to be a more significant predictor of both poor sleep quality and insomnia than other symptoms such as pain, cough and heartburn [200].

Nocturia may be caused by benign prostatic hypertrophy (BPH), primary or secondary detrusor overactivity and pathologies associated with impaired bladder contractility [163]. In addition, excess fluid intake, diabetes mellitus, diabetes insipidus and hypercalcaemia are associated with nocturia [159]. It has also been seen in HF, HTN, venous stasis disorders, hypoalbuminemia, hepatic and renal failure [159,163]. This may be partially due to medications, but may also be due to atrial natriuretic peptide (ANP) and the movement of third spaced fluid of the lower extremities when patients are supine [163]. Sleep disorders and nocturia are also associated [201]. Medications associated with nocturia include diuretics, antihypertensives such as calcium channel blockers and sodium-glucose co-transporter-2 (SGLT2) inhibitors [202,203].

Determining the underlying mechanism of nocturia is the first step in management. Detailed history taking and examination is recommended. Investigations, when indicated, may include frequency volume charts, urinalysis, serum analysis (including HbA1c, calcium, renal function, prostate-specific antigen) and measurement of post-void residual volume (PVR) [159]. Specialist referrals and cystoscopy may be required, and treatment should be tailored to the underlying cause. Lifestyle measures may be helpful, including reducing consumption of nocturnal fluid and diuretic intake, measures to prevent and reduce pedal oedema and consideration of timing, classes and formulation of medications [204,205]. Specific treatments, such as alpha 1 receptor antagonists and 5 alpha reductase inhibitors for BPH, and bladder relaxants for those without a high PVR and symptoms suggestive of detrusor overactivity, may be helpful, while desmopressin should be considered with absolute caution due to the risk profile in older patients [204]. Given that shorter time to first void has been associated with poorer sleep quality, increasing time to first void may be helpful for sleep if nocturia cannot be eliminated [206,207].

### 4.7. Parkinson’s Disease

It has been postulated that sleep and circadian dysfunction may partially drive neurodegeneration in the early phases of PD, and that neurodegeneration may also negatively impact sleep, with a potential self-perpetuating cycle in PD [208,209]. Sleep disturbance is commonly seen in the prodromal phase of PD, prior to motor symptoms developing [210,211,212]. Good mobility on waking (sleep benefits) have also been observed in PD patients, and poor sleep has been associated with dyskinesia [213,214].

Impaired sleep in PD is likely multifactorial, related to neurodegeneration, underlying sleep disorders, PD symptoms and treatment. Nocturia is common in PD, and negatively impacts sleep continuity [215,216]. Nocturnal motor symptoms of PD may also contribute to impaired sleep, in addition to medications for PD, pain and depression [217,218].

Treatment of sleep disruption in PD is complex. Exacerbating factors and whether underlying sleep disorders are present should be considered and treated as appropriate, with both insomnia and RBD being common in PD [219,220]. Careful consideration of PD treatment may also be of benefit. Both selegiline and amantadine, for example, have stimulant effects, and should be taken earlier in the day [221]. Excessive nocturnal dopaminergic stimulation may also result in reduced total sleep time [222,223]. This should be balanced against nocturnal motor symptoms and their effect on sleep. Levodopa-carbidopa controlled release may improve nocturnal motor symptoms that interfere with sleep, but this has not shown consistent significant improvements in sleep parameters [222,224]. Dopamine agonists have shown some benefit in relation to sleep, as a secondary endpoint in several RCTs [225]. Rotigotine’s effect on sleep has been observed in small dedicated trials, with statistically significant positive effects on sleep measures [226,227]. The monoamine oxidase B inhibitor, rasagiline, as an addition to Levodopa treatment may also be beneficial in relation to sleep [228]. Hypnotics should, again, be considered with absolute caution, given their risk profile in the older population, in addition to insufficient evidence in the PD population in relation to efficacy [225,229]. Small studies of melatonin use in PD have shown promising results, and this may be due to circadian dysfunction in PD patients [230,231,232].

### 4.8. Dementia

There also appears to be a bi-directional relationship between dementia and sleep, with sleep disruption representing both a risk factor for, and symptom of, the neurocognitive syndrome [233,234,235]. A recent systematic review and metanalysis found that sleep problems, including both short and long sleep duration, insomnia, OSA, impaired circadian rhythm and sleep quality, were associated with an increased relative risk of preclinical AD, cognitive impairment and AD [236]. Sleep disturbance also occurs commonly in AD and other forms of dementia [237]. Patients with AD can have increased sleep onset latency, and reduced time spent in restorative SWS and REM sleep [238]. Sleep disturbance may be even more common in DLB, where there is an aforementioned association with RBD [239]. Disturbed sleep may also impact carer burden and carer sleep, QOL, and is associated with a higher risk of institutionalisation [240,241,242,243].

Evidence is lacking for many commonly used medications for sleep disturbance in dementia [244]. In some cases, melatonin may be beneficial, and it may aid nocturnal behavioural and psychological symptoms of dementia (BPSD), alongside non-pharmacological interventions (which should be first line) [245,246]. If pharmacological intervention is deemed necessary for sleep disturbance and BPSD, trazadone may also be effective [247].

Given the risk associated with hypnotics, non-pharmacological management has an important role [229]. Evidence in this area was recently explored by O’Caoimh et al. in a systematic review [248]. Meta-analysis of RCTs showed a statistically significant improvement in sleep efficiency in intervention vs. control participants, but no significant difference in total sleep time, number of night-time awakenings or day time sleep duration. Looking at individual non-pharmacological categories, multi-domain interventions appeared to be associated with statistically significant improved sleep efficiency, while there was insufficient evidence to support the efficacy of light therapy. There was only one study looking at CBT-I. Patients with mild cognitive impairment (MCI) living in residential care were randomly assigned to six CBT-I sessions, or active control, and improved sleep was observed in the CBT-I group [249]. A further RCT, published following this systematic review, looked at dCBT-I in participants with insomnia and memory or attention complaints, but not dementia or MCI, and showed improved cognitive and sleep outcomes in the intervention arm [250].

### 4.9. Medications and Polypharmacy

Commonly prescribed medications in the context of co-morbidity may also negatively impact sleep. Antihypertensives, antidepressants, antiepileptics, corticosteroids, decongestants and diuretics are among many medications that have been associated with insomnia, and may represent potentially inappropriate medications in the older patient [251,252,253,254]. The number of potentially inappropriate prescribed medications has also been associated with reduced sleep efficiency and sleep quality [255]. In addition, in a recent longitudinal study, patients who underwent poly-deprescribing saw significant improvements in subjective sleep quality vs. those who did not [256]. Reviewing current and recently changed medications that can contribute to sleep symptoms may be helpful in patients with sleep complaints [49]. This should also take into account the complex picture of co-morbidity management, QOL and patient preference, with particular attention to potentially inappropriate prescriptions.

### 4.10. Falls

Sleep disturbance has also been associated with falls, a major cause of morbidity and mortality in older patients [9,257,258]. This association appears to hold true even after adjusting for the use of hypnotics and psychotropic medications [259,260,261,262]. Increased risk of hip fractures has also been seen in patients who have sleep disturbance, with increased incidence of injurious falls seen in self-reported short and long sleep duration [263,264]. To the author’s knowledge, there have been no large RCTs looking at non-pharmacological interventions to improve sleep and outcomes of falls; however, a recent multicomponent, patient-centred, phone-based RCT, with “better sleep” as one of four modules, demonstrated reduced falls and fractures in the intervention arm [265]. This is an area where further research is merited, given that impaired attention is associated with sleep impairment and falls, and nocturnal awakenings are associated with falls, non-pharmacological interventions to improve sleep may have a significant impact on the risk of falls in the geriatric population [266,267,268,269].

### 4.11. Other Co-Morbidities

Sleep disturbance and disorders have also been associated with many other co-morbidities, many in potential bi-directional relationships. These include HTN, diabetes, obesity, stroke, renal disease and cancer, among others [270,271,272,273,274,275]. Exploration of further co-morbidities, however, is beyond the scope of this review. Considering non-pharmacological options and optimising co-morbidity management, with cognisance of medications and interventions that can exacerbate sleep problems, is likely a reasonable general approach.

## 5. General Approach and Management of Older Persons with Sleep Complaints

The author suggests that assessment of sleep health, and further assessment where warranted, form part of every comprehensive geriatric assessment (CGA). Although there has been little validation in the older population, screening and assessment tools can be considered, with some screening and assessment tools explored in Table 2. If there appears to be an issue with sleep on initial assessment, the author suggests a systematic approach. Older patients are a heterogenous group and may suffer from several different sleep disorders, co-morbid conditions and other factors affecting sleep. Ascertaining the type of sleep issues experienced, other attendant symptoms, co-morbidities, medications, psychosocial and environmental factors are key elements of assessment [27]. Particular information should be obtained in relation to:

- Snoring or apnoeic episodes at night;
- Abnormal movement during sleep;
- Restless leg symptoms;
- Nocturia;
- Medications and comorbidities;
- Pain;
- Psychological symptoms;
- Social circumstances;
- Sleep hygiene issues i.e., caffeine consumption, routine, light exposure, etc.

**Figure 1:**
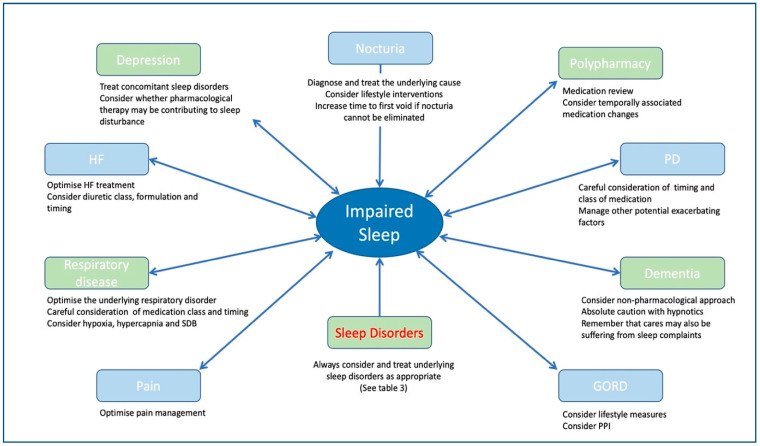
Summary of co-morbidities and considerations in the context of sleep complaints. HF = heart failure, GORD = gastro-oesophageal reflux disease, PD = Parkinson’s disease, SDB = sleep disordered breathing.

Furthermore, focused physical exams and investigations may be necessary, including referrals for PSG. Assessment-based treatment for particular sleep disorders may be indicated in addition to optimising underlying co-morbidities, pain, addressing exacerbating medications, psychosocial factors and sleep hygiene [27]. A synopsis of common sleep disorder diagnosis and management and co-morbidity considerations can be found in Table 3 and Figure 1, respectively. Hypnotics, again, should only be used with absolute caution, for brief periods if thought required, and consideration of non-pharmacological options is essential.

## 6. Conclusions

Sleep complaints are both common and complex in the older person. Their consideration is an important aspect of holistic care, and may have an impact on QOL, mortality, falls and disease risk. Considering sleep disorders, co-morbidity, medication management and non-pharmacological options are important aspects of a multifaceted approach in this group. The author provides a brief overview of sleep disturbance in the context of sleep disorders and several co-morbidities, and a suggested general assessment approach that may be helpful in this complex task.

## Figures and Tables

**Table 1:** Examples of intrinsic and extrinsic factors that may negatively impact sleep in older patients.

Intrinsic Factors	Extrinsic Factors
Changes in the ageing brain	Life events
Changes in hormone secretion	Physical activity
Changes to the lens and retina	Social engagement
Cumulative co-morbidity	Medications

**Table 2:** Examples of sleep assessment/screening tools and their utility in older patients.

Tool:	Purpose:	Studied in Older Populations:	Comments/Limitations:
Pittsburgh Sleep Quality Index (PSQI) [276]	Investigates sleep quality and disturbance.	Yes, with reasonable validity [276,277,278].	Can take ≥30 min to complete and requires recollections from the previous month, so it may not be appropriate for a geriatric clinic where sleep is one of many issues being addressed, or where patients suffer short-term memory problems [279].
Epworth Sleepiness Scale (ESS) [280]	A tool to profile excessive daytime sleepiness. High scores of >10 are less common in insomnia and should trigger further interrogation for another sleep disorder [279].	Yes, with reasonable validity [278].	Some geriatric populations may find the scale difficult to complete [281].
Insomnia Severity Index (ISI) [282]	Instrument to assess insomnia severity.	Some evidence to suggest validity in the older population [283,284].	Applies to insomnia only and does not assess sleep generally or symptoms that may be relevant to other sleep disorders [285].
Patient-Reported Outcomes Information System (PROMIS) Sleep Disturbance Scale [286]	The six-item scale assesses perceptions of sleep quality, restfulness, sleep problems and difficulty falling asleep.	Some evidence to suggest validity of the six-item scale in the older population [285].	Requires recollections from the past 7 days and does not assess symptoms of specific sleep disorders, but the overall severity of sleep problems.
The Essener Questionnaire on Age and Sleepiness (EQAS) [287]	Assessment of observed daytime sleepiness.	Some evidence to suggest validity, with low participant numbers [287].	Can be filled out by carers following patient observation, and may be an option in patients with cognitive impairment or communication difficulties, although it does not assess for multiple sleep disorders.
Berlin Questionnaire (BQ) [288]	Identify patients at risk of OSA.	Studied in an exclusively older population, and was found to have reduced accuracy to discriminate between those with and without OSA [289].	May have limited discriminative utility in older patients, and screens for OSA alone.
STOP-BANG questionnaire [290]	Identify patients at risk of OSA.	Recently studied in an exclusively older population found to be of limited utility [291]. A very high proportion met OSA criteria, and an argument against screening in this population was made, with further research needed.	May have limited discriminative utility in older patients, and screens for OSA alone.
Sleep Apnoea Clinical Score (SACS) [292]	Identify patients at risk of OSA.	Studied in a cohort of COPD patients where mean age was >65, with reasonable predictive ability in comparison to ESS and BQ [293].	May be an option in older patients with COPD, although further studies are needed.

For restless leg syndrome and REM sleep behaviour disorder, single screening questions should be considered (see dedicated sections). BQ = Berlin Questionnaire, ESS = Epworth Sleepiness Scale, EQAS = The Essener Questionnaire on Age and Sleepiness, ISI = Insomnia Severity Index, OSA = obstructive sleep apnoea, PSQI = Pittsburgh Sleep Quality Index, PROMIS = Patient-Reported Outcomes Information System, SACS = Sleep Apnoea Clinical Score.

**Table 3:** Synopsis of common sleep disorder diagnoses and management in the older patient.

Sleep Disorder	Diagnosis	Non-Pharmacological Options	Pharmacological Options
Insomnia	Clinical history Sleep questionnaires/tools and PSG are supportive	CBT-I dCBT-I SHE Relaxation Mindfulness	Second-line/short-term Dependent on patient
Sleep Disordered Breathing	PSG Portable home-based devices	PAP Weight loss where appropriate	Consider reduction in sedating medications
Restless Leg Syndrome	Clinical history Screening question may be helpful	Limited evidence	Iron replacement in deficiency Dopamine agonists Alpha-2 delta calcium channel ligands Levodopa Benzodiazepines and opioids with caution
REM sleep behaviour disorder	PSG Screening question and collateral may be helpful	Modifying sleeping environment if concerns for injury	Review for potential exacerbating medications Limited evidence for melatonin and clonazepam Consider potential exacerbating medications

CBT-I = cognitive behavioural therapy for insomnia, dCBT-I = digital cognitive behavioural therapy for insomnia, PAP = positive airway pressure therapy, PSG = polysomnography, SHE = sleep hygiene education.
